# Acute cough in Italian children: parents’ beliefs, approach to treatment, and the family impact

**DOI:** 10.1186/s40248-019-0180-9

**Published:** 2019-04-04

**Authors:** Roberto W. Dal Negro, Alessandro Zanasi, Paola Turco, Massimiliano Povero

**Affiliations:** 1 National Centre for Respiratory Pharmacoeconomics and Pharmacoepidemiology, Verona, Italy; 2 Italian Association for Cough Study (AIST), Bologna, Italy; 3 Research & Clinical Governance, Verona, Italy; 4 AdRes Health Economics and Outcome Research, Torino, Italy

**Keywords:** Acute cough, Acute cough in children, Parents’ beliefs, Prescribing attitude, Cough impact

## Abstract

**Background:**

Acute cough is the most common symptom among children in primary care, but the impact of cough episodes was never investigated in Italian families.

**Methods:**

A cross-sectional telephone survey was conducted on a representative sample of Italian families, randomly selected from general population; a specific and validated questionnaire was used.

**Results:**

The sample (604 calls) was uniform by geographical distribution, and by children age and gender. Mean cough episode was 3.1/year, they were short lasting (only 4.7% > 2 weeks). Independent predictors of children cough episodes were parents’ active smoking habit and work (*p* < 0.05). The mean nursery/school absenteeism was mostly < 7 days, but of a 7–15-day duration in near 30% of cases. The pediatrician was contacted immediately only by 25% of parents and a second consultation (mostly a lung physician) usually occurred after 2–3 weeks of cough. Meanwhile, home/pharmacist suggested remedies were adopted in 50–70% of cases. Usual prescriptions were mucolytics (85.8%), antitussive agents (55.6%), non-steroideal anti-inflammatory drugs (33.8%), antibiotics (regularly or episodically 80%), and corticosteroids (systemic steroids in less than 50%, but via aerosol in more than 80% of cases). Moreover, pediatricians claimed to use homeopathic drugs regularly or episodically in almost 50%. The respondents’ willingness to spend out-of-pocket for an “effective remedy” against cough was of € 20 (>€ 30 in 18.4% of cases).

**Conclusions:**

Parents’ actions against cough episodes were variable, depending on their beliefs, smoking habit, and occupational status. The parents’ perceived efficacy of usual prescriptions is poor, and their willingness to pay out-of-pocket for an “effective remedy” against cough is high. The interest for alternative treatments is not negligible in these circumstances.

**Electronic supplementary material:**

The online version of this article (10.1186/s40248-019-0180-9) contains supplementary material, which is available to authorized users.

## Introduction

Cough is a physiological mechanism that allows both the protection from the inhalation of airborne irritant materials and the function of clearing secretions from the airways [1]. Nevertheless, cough also represents one of the most common clinical problems, being a non-specific symptom which can be related to a large number of diseases such as bronchial asthma, allergic disorders, post-nasal drip, upper airway infections.

Cough can be acute or chronic (more than 8 weeks) disorder; acute cough is the most common symptom among children in primary care, and it is mostly due to seasonal respiratory infectious events [2–6].

Even if acute episodes caused by simple cold usually tend progressively to resolve spontaneously within 4–5 days, cough episodes due to influenza and upper airway infections are more severe even in the presence of a mild infection [7–9]. Further to inducing a huge number of general practitioner (GP) or pediatrician visits every year, these episodes represent a relevant cause of school absenteeism, which in turn can also affect parents’ working activities substantially [10]. For these reasons, high rates of vaccination coverage are encouraged and promoted because these actions can minimize both the morbidity and the societal impact of airway tract infections, together to the impact of related cough episodes [11].

The epidemiology of cough was only episodically investigated in Italian children [12], but the true impact of cough episodes on their families was never assessed in Italian general population to our knowledge.

Aim of the study was to investigate the clinical characteristics of cough episodes in children, parents’ beliefs, and pediatrician’s attitude to therapeutic intervention.

## Material and methods

The study was planned by National Centre for Respiratory Pharmacoeconomics and Pharmacoepidemiology (CESFAR), the Italian Association for Study of Cough (AIST), and AdRes. The study (conducted between March 5^th^- 21^st^, 2017) was approved by the CESFAR Ethical Committee on October 26^th^, 2016 (n.A/002/16).

A cross-sectional telephone survey was carried out on a representative sample of Italian families with children, randomly selected from general population. The tool for investigation was a specific, validated, anonymous questionnaire (Additional file 1). All interviews to children’s parents were conducted according to the Computer Assisted Telephone Interview (CATI) methodology [13] by expert, professional interviewers. The interviewer was provided with one “work station” consisting of a personal computer connected to a central processing unit. The central unit was also equipped with a specific software for the random choice of individuals (such as, the telephone numbers) to contact. The sampling strategy adopted in the present survey was the random selection of an adequate number of subjects. All interviews were always preceded by a short explanation of the aim of the survey, and had a mean duration of ten minutes.

A minimum number of 600 respondent families with children was previously calculated for achieving the representativity of the sample in terms children’s age, gender, and geographical distribution (3% mean effect size; 5% significance level, and 80% statistical power) [14].

Only interviews obtained after obtaining the respondent’s informed consent to the interview and to the possible use of information for scientific purposes were considered.

### Statistical analysis

All collected data were summarized using percentage of responses, or mean and standard deviation. For continuous outcomes not normally distributed, median and interquartile range were also reported. As concerning categorical answers, the mean value of each category was considered in order to compute the weighted mean.

Normality was tested using Shapiro-test while differences in categorical outcomes were assessed using Chi-squared test; a *p*- lower than 0.05 was chosen to asses statistical significance. All analyses were performed using computer software R 3.1.2 [15].

## Results

Total calls were 1,716, and only 79 contacts (4.1%) refused immediately their consensus. A total of 604 questionnaires were usable, and then collected and analyzed, by a redemption rate of 35.2%.

Respondent parents had an average age of around 40 years (IQR: 36–44) and their children of about 7 years (IQR: 4–9). The distribution of responders proved absolutely uniform in geographical terms and also by their child/children’s age and gender. In the majority of cases, mothers (80%) filled questionnaires; furthermore, at least one of the two parents was an active smoker in almost 60% of respondent families (Table 1).

**Table 1:** General characteristics of children and families

	Mean (SD)	Median (IQR)
Age of child	6.92 (3.28)	6 (4─9)
Age of respondent parent	40.39 (6.19)	41 (36─44)
Gender of child (% male)	52.98%	
Gender of respondent parent (% female)	80.46%	
Smokers in the family (% at least un parent)	57.57%	
Geographic area		
North-West	25.17%	
North-Est	25.17%	
Center	21.52%	
South and Islands	28.15%	

*SD* standard deviation, *IQR* interquartile range

The joint distribution by parents’ job is reported in Table 2: clerk-clerk, clerk-laborer, clerk-housewife, laborer-housewife, and laborer-laborer were the most frequent job combinations within the parents’ sample.

**Table 2:** Joint distribution of both parents by their occupation

Parent 2	Entrepreneur	Manager	Clerk	Laborer	Craftsman	Self employed	Merchant	Independent contractor	Retired	Housewife	Unemployed
Parent 1											
Entrepreneur	0.2%										
Manager	0.2%	0.7%									
Clerk	1.0%	2.0%	21.0%								
Laborer	0.3%	0.7%	13.4%	4.9%							
Craftsman	0.3%	0.0%	0.7%	0.7%	0.2%						
Self employed	0.7%	0.3%	7.9%	3.4%	0.5%	1.3%					
Merchant	0.3%	0.0%	1.2%	0.5%	0.0%	0.7%	0.0%				
Independent contractor	0.3%	0.2%	1.5%	0.5%	0.2%	1.2%	0.3%	0.3%			
Precarious worker / student	0.0%	0.0%	0.3%	0.0%	0.0%	0.2%	0.0%	0.0%	0.0%		
Housewife	0.3%	0.8%	8.1%	6.7%	0.3%	3.4%	0.2%	0.5%	0.2%	0.8%	
Unemployed	0.3%	0.2%	4.2%	2.9%	0.2%	1.7%	0.2%	0.2%	0.0%	0.3%	0.3%

In the last 12 months, more than 90% of children experienced cough episode: more than half of these children had at least 3 cough episodes and 13.4% more than five episodes over the same period (Table 3). The mean annual rate results in 3.15 episodes per year. Usually, these episodes had a short duration, and cough lasted for more than 2 weeks only in 5.4% of cases.

**Table 3:** Rate and duration of cough episodes

	Total population	Parents’ smoking status			Geographical distribution			
		Both smoking	One smoking	None smokers	North-West	North-Est	Center	South and Islands
% child with cough episodes	91.06%	95.92%	90.81%	90.56%	89.47%	94.74%	91.54%	88.82%
Number of episodes (mean episodes per year)	3.15	3.34	3.14	3.13	3.03	3.25	3.13	3.17
1–2 episodes	44.91%	38.30%	42.70%	46.96%	47.79%	42.36%	45.38%	44.37%
3–5 episodes	41.64%	46.81%	47.03%	37.70%	41.18%	42.36%	41.18%	41.72%
> 5 episodes^a^	13.45%	14.89%	10.27%	15.34%	11.03%	15.28%	13.45%	13.91%
Length of episodes (mean days per episode)	8.42	9.06	8.50	8.27	9.15	8.22	8.36	8.02
< 7 days	40.91%	31.91%	39.46%	43.45%	35.29%	40.28%	44.54%	43.71%
7–15 days	53.64%	61.70%	55.68%	50.80%	56.62%	56.25%	49.58%	51.66%
16–30 days	4.73%	6.38%	3.78%	5.11%	7.35%	2.78%	4.20%	4.64%
> 30 days	0.73%	0.00%	1.08%	0.64%	0.74%	0.69%	1.68%	0.00%

^a^Maximum number of episodes per year is 7. This limit was estimated fitting a Poisson distribution on the frequencies recorded in the questionnaires

While children suffering from 1 to 2 cough episodes/year were much more frequent in smoke-free families (Table 3), those suffering from 3 to 5 episodes/year were significantly more frequent in families where at least one parent was an active smoker (*p* < 0.046). Also the 7–15-day duration of cough episodes was more frequent in families with active smokers when compared to that of the smoke-free ones (such as: a prevalence of 61.7% vs 50.8%, respectively). Unfortunately, even if pretty clear, this tendency did not reach the minimum level of statistical significance.

**Fig. 1:**
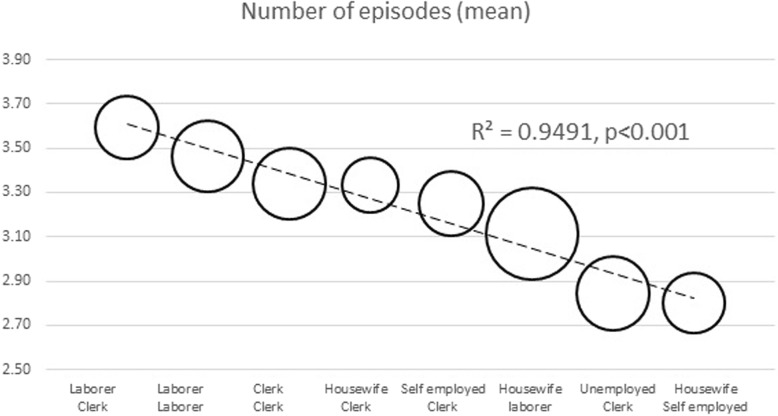
Relationship between the parent’s employment and the mean rate of cough episodes (chi-square test)

The geographical distribution of families did not affect neither the rate nor the duration of cough episodes (Table 3). On the contrary, the rate of cough episodes proved affected by the parents’ occupational status; it was significantly higher in the families where both parents were active workers, and significantly lower in those families where at least one parent was at home for the great part of the day (Fig. 1).

In the majority of cases cough was reported as initially dry, and accompanied by secretions of variable extent in the following days, while dry cough only and cough immediately productive were reported in a small proportion of case. In the large majority of cases cough was lasting over the day, followed by over day and night (Table 4).

**Table 4:** Type and occurrence of cough

Type	
Dry	14.55%
Initially dry followed by secretions	74.18%
Initially with secretions	10.55%
Don’t know / prefer not to answer	0.73%
Occurrence	
Day only	11.82%
Day and night	82.36%
Night only	5.27%
Exertional only	0.55%

Cough episodes affected both children’s sleep and school activities at different extent in the large majority of cases (Table 5). Even if the mean nursery/school absenteeism was generally shorter than 7 days, in about 30% of cases duration is 7–15-day. One of the two parents (69.6%), or grandparents (29.1%), usually took care of the child/children, resulting the baby-sitter or other caregiver’s role quite negligible in the present study.

**Table 5:** Effect of cough episodes on both sleep and school activities

	Sleep	School activities	Nursery/school absenteeism		Home caring	
Never	3.31%	12.91%	< 7 days	62.73%	Parent(s)	69.60%
Sometimes	63.91%	59.27%	7–15 days	28.36%	Grandparents	29.12%
Often	25.83%	22.85%	16–30 days	3.64%	Other^a^	1.28%
Always	6.95%	4.47%	> 30 days	4.55%		
Don’t know/prefer not to answer	–	0.50%	No kindergarten/school	0.73%		

^a^including baby-sitter

When a cough episode occurred, the medical referral was usually rapid: 26% of parents tended to contact the pediatrician immediately, while in the majority of cases the doctor was called within the first 7 days of child’s cough. A second referral (of a lung physician in the majority of cases) usually occurred when cough did not disappear or attenuate significantly within 2–3 weeks (Table 6). Meanwhile, home remedies or symptomatic remedies suggested by the pharmacist were usually adopted in 71.5 and 51.2% of cases, respectively.

**Table 6:** Time for a pediatrician and of another specialist consultation

	Pediatrician	Another specialist
Immediately	25.50%	2.32%
< 7 days	60.60%	25.99%
7–15 days	8.77%	30.63%
16–30 days	2.32%	19.04%
> 30 days	0.83%	9.60%
Don’t know / prefer not to answer	1.99%	12.42%

The pediatrician’s therapeutic approach to cough is summarized in Table 7. In general, the aerosol therapy appeared the preferred route for drug administration in these circumstances. Systemic antitussive and non-steroideal anti-inflammatory drugs were also frequently prescribed as first-line options (such as, in more than 25% of cases). Antibiotics were not used in near 15% of cases only, but they were prescribed regularly or episodically in near 80% of cases. Systemic corticosteroids were reported as episodically prescribed in near 50% of episodes, but usually prescribed in 5% of cases. However, mucolytics (85.8%), cough suppressant (55.6%), and non-steroideal anti-inflammatory drugs (33.8%) were the most preferred therapeutic options.

**Table 7:** Pediatrician’s most prescribed drugs and parents’ concerned toward prescriptions

	Antibiotics	Corticosteroids	Antitussive Anti-inflammatory	Aerosol
Prescribed by pediatrician				
Always	5.30%	4.97%	26.66%	33.61%
Sometimes	78.15%	43.38%	64.07%	49.34%
Never	15.40%	49.67%	7.95%	17.05%
Don’t know / prefer not to answer	1.16%	1.99%	1.32%	–
Parents’ concerned				
No	8.33%	5.48%		
A little	36.51%	26.37%		
Very	47.02%	46.23%		
Highly	8.13%	20.55%		
Don’t know / prefer not to answer	–	1.37%		

The general respondents’ concern was more or less equally distributed against corticosteroids and antibiotics, even if their highest concern was against corticosteroids rather than antibiotics (20.6% vs 8.1%, respectively) (Table 7). In general, these drugs were perceived as effective by 48.7%, and highly effective by 4.8% of respondents, even if in 36.8% of cases the effectiveness was reported as poor, and in 7.1% they were defined as ineffective.

The respondents’ willingness to spend out-of-pocket for an “effective remedy” against their children’s cough ranged € 14.0–25.9, by an average of € 20.0. Even if their willingness to spend was of € 10–20 in more than 53% of cases, it ranged € 20–30 in 10.7%, and was higher than € 30 in 18.4% of cases, respectively (Table 8).

**Table 8:** Willingness to pay for an effective cough product

Mean (range)	€ 19.95 (14.04─25.86)
Nothing	0.33%
< 10 euros	10.43%
10–20 euros	53.64%
20–30 euros	10.76%
> 30 euros	18.38%
Don’t know / prefer not to answer	6.46%

Finally, while 51.7% of pediatricians never prescribed homeopathic drugs against cough (at least according to parents’ interview), 24.7% of them prescribed these drugs regularly, and 23.7% episodically (Table 9). Homeopathic anti-cough drugs were defined effective by 34.8%, and not or poor effective by 53.6% of respondents, respectively. When the reasons of their beliefs were specifically requested, almost 65% of pro respondents claimed that homeopathy would be regarded as a safe and alternative therapeutic option in these cases. On the contrary, more than 35% of on respondents were adamant in denying any efficacy to these drugs.

**Table 9:** Parents’ beliefs on homeopathic drugs

	Yes	No
Homeopathy as an alternative	64.72%	35.28%
Why yes?		
Safe	57.88%	
Effective	26.97%	
Alternative	15.15%	
Why not?		
Non effective		84.02%
Less effective		11.24%
Too much expensive		4.73%

## Discussion

In concordance with consolidated literature [2–6, 8, 9], the incidence of cough episodes reported in present survey proved very high indeed, as more than 90% of Italian children suffered from these events over the year, though at different rates, severity, and duration.

These events (mostly due to upper respiratory tract infections of viral origin) cause significant, even though transient, limitations in the usual activities and in the quality of life of sick children, but can also frequently cause the involvement of other family members. It is then presumable that this sort of epidemic spreading within the family would have further triggered a not negligible limitation also in parents’ daily life, particularly when parents are the only home care-givers of their child/children.

Furthermore, it should be considered that cough episodes occur much more frequently in a sizable cluster of children than in others, and the overall duration of all the annual events can lead to a relevant socio-economic impact in these cases.

The parents’ smoking habit plays a quite relevant role, as this negative attitude proved strictly related to the annual rate of children’s cough episodes in the present study, independently of the geographical region where the family lives. Even if only acute cough was investigated in the present study, data further support, even if indirectly, the dangerous effect of the indoor second-hand smoke on children [16] and strongly stress the heavy responsibility of smoking parents versus their own children.

Moreover, the strict relationship between the status of parents’ occupation and the rate of annual cough episodes came out from the present study as a quite novel piece of evidence. Actually, data seem to suggest that when at least one parent is usually at home for many hours/day, the impact of cough episodes is significantly lower. Several factors can be suggested for explaining this phenomenon: in particular, a reduced infectious risk and a more effective home children caring can be presumed in these cases. Conversely, the child caring provided by other care-givers appears less effective, also in terms of cough episode duration. In the particular case of grand-parents’ caring, it also plausible that their emotional concern may lead to the magnified description of cough events.

In terms of children’s home management, two main factors are presumed to affect the scenario in these circumstances: first, the parents’ desire to define and resolve the cause of symptoms of their child/children as soon as possible, and, secondly, the parents’ need to safeguard their job and their economic position. As suggested in previous papers [17, 18], both these conditions contributes to stimulate a quick doctor’s intervention.

Actually, even if domestic remedies and those suggested by the pharmacist are generally immediately preferred, the medical referral is usually searched with a very short delay since the cough onset. Criteria which lead to the doctor consultation are of several origins, and not fully understood yet. The persistency of intense cough for 24 h, together with a persisting fever, and the occurrence of other respiratory signs (namely, dyspnea and/or wheezing) are presumed to represent the major determinants [19]. However, other factors can further differently contribute to the parents’ concern and to the decision in favor of a medical intervention, such as: the anxiety of one or of both parents; their insufficient coping skills (particularly if they are young and at their first experience); the role of other family components (mainly grand-parents) who press for a rapid diagnosis and a rapid therapeutic intervention; the disruption of their daily organization; the fear of negative effects on their working life [20, 21].

As expected, the pediatrician is usually the first doctor to be consulted, followed by the Lung Physician, who is preferred in particular clinical conditions, such as when cough is long-lasting or is complicated by other respiratory signs (namely, wheezing; at rest or exertional dyspnea, high fever, etc.).

The vast majority of pediatricians’ home prescriptions consists in antibiotics, corticosteroid (mostly via the inhalation route), mucolytics, antitussive drugs, and non-steroideal anti-inflammatory agents. As in previous studies [22, 23], antibiotics still result over-prescribed and their use is presumed as largely inappropriate also in the present survey. Actually, the reported characteristics of cough (initially mainly dry for a few days) would have suggested the occurrence of an upper airway viral infection in the majority of cases. Consequently, the immediate prescription of antibiotics and/or oral steroids should then have been regarded as an inappropriate first-line therapeutic choice. Nevertheless, data from the present survey further confirm how persisting is the attitude of antibiotic prescribing in primary care, independently of the subjects’ age and of a precise etiology of cough [24, 25], and regardless of the high parental concern against antibiotics and corticosteroids.

From this point of view, it should be emphasized that also the National Guidelines for the management of influenza-like syndrome in adults and children do not recommend the use of antibiotics in non-complicated conditions, unless the clinical picture is evident for proven bacterial co-infections [26]. Moreover, a recent Cochrane review investigated the benefits and the risk of antibiotics for cough due to acute bronchitis, being cough one of the most common disorders to face in primary care. Results confirmed that there is limited evidence in favor of the antibiotics use in these circumstances. However, the magnitude of benefits achievable should be further carefully compared to the potential side effects and to the risk of increasing induced-resistance to respiratory pathogens [27].

As reported above, the aerosol route for drug administration was largely preferred by pediatricians, even if there still is insufficient evidence for recommending inhaled steroids in cough due to upper respiratory tract infections [28], and several studies recommended the reduction of “over-the-counter” anti-cough medicines in children due to their several side-effects, particularly during the first years of life [29, 30].

In general, the concept suggested in some previous papers that “the cure can be worse than the cough” in several cases of primary care management [31–33] is then plausible and worth to be shared in the majority of cases.

Actually, the care-givers’ perceived efficacy of usually prescribed drugs is generally poor during cough episodes. This evidence is also confirmed by the high parents’ willingness to pay out-of-pocket for an “effective remedy” against their child’s cough. A further indirect confirmation of this hypothesis comes from the positive attitude of respondents versus alternative therapeutic interventions against cough. At present, this complementary approach results in increasing progression in our as in other countries [34]. However, this approach is still generally “belief-based”, and further controlled scientific information is needed for pediatricians and families, particularly in terms of potential side effects and risk.

The present study has some limitations: it was conducted according to a cross-sectional, retrospective design, not aimed to the precise definition of cough etiology and to the assessment of prospectic outcomes. Information were collected by a questionnaire also usable by care-givers different from the two parents. Furthermore, information on therapeutic prescriptions were derived from parents or care-givers, and not directly from pediatricians or GPs.

A point of strength is that the study was conducted on a representative sample of Italian families evenly distributed across the national regions, and the corresponding redemption was pretty good.

## Conclusions

Cough is very frequent in children. Cough episodes recognize variable etiology, rates, and duration over the year. Both the rate and the duration of cough episodes can cause a substantial impact on families, depending of their occupational status and smoking habit. The parents’ behavior when facing their children’s cough episodes is variable and depending of several factors, still not explored exhaustively. Their job status, smoking habit and family organization affect decisions. Usual home prescriptions are largely inappropriate and frequently merely related to environmental suggestions. The parents’ perceived efficacy of usual therapeutic options is frequently poor and, conversely, high is the willingness to pay out-of-pocket for an effective remedy against cough. The interest for alternative treatments is also not negligible in these circumstances.

## Additional file

Additional file 1: Cough Questionnaire. (DOCX 118 kb)
