# Supplementary figures and images for: Acute cough in Italian children: parents’ beliefs, approach to treatment, and the family impact
Source: Multidiscip Respir Med. 2019 Apr 4;14:16. doi: 10.1186/s40248-019-0180-9 (PMC6448239; doi:10.1186/s40248-019-0180-9)

# **Additional file 1**


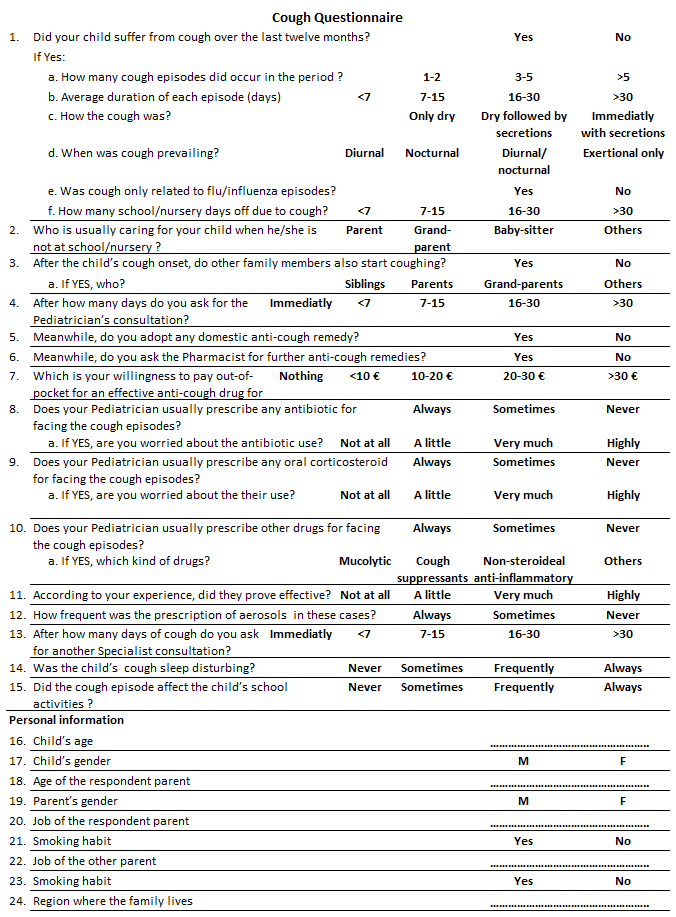

Supplement: Supplementary file 1 — Cough Questionnaire. (DOCX 118 kb) [file 40248_2019_180_MOESM1_ESM.docx]
